# Navigating the Emotional Challenges of Grade School: The Influence of Early Family Strains and a Lack of Parental Responsiveness on Trajectories of Anxiety and Depressive Symptoms

**DOI:** 10.1111/famp.70101

**Published:** 2025-12-25

**Authors:** Marla Lopez, Rebecca L. Brock, Tiffany D. James, Jennifer Mize Nelson, Kimberly Andrews Espy, Timothy D. Nelson

**Affiliations:** ^1^ Department of Psychology University of Nebraska‐Lincoln Lincoln Nebraska USA; ^2^ Office of Research and Economic Development University of Nebraska‐Lincoln Lincoln Nebraska USA; ^3^ Department of Psychiatry and Behavioral Neurosciences Wayne State University School of Medicine Detroit Michigan USA

**Keywords:** child negative emotionality, family strains, internalizing symptoms, responsive parenting

## Abstract

Although parenting is critical to children's emotional development, broader family processes can also play an important role in the development of anxiety and depressive symptoms in children. The current study examined the influence of early family strains (i.e., chronic stressors and resource deficits) on children's trajectories of anxiety and depressive symptoms throughout grades 1–4 via (less) responsive parenting at preschool age. Further, we examined whether familial factors were differentially associated with child anxiety and depressive symptoms as a function of child temperamental negative affect, a prominent risk factor for internalizing problems. In a US longitudinal study including five waves of data spanning preschool to grade 4 (*N* = 496), analyses revealed that higher levels of family strains were associated with less responsive parenting during preschool, *b* = −0.16, *p* = 0.001. Less responsive parenting was associated with greater escalation in anxiety and depressive symptoms over grade school for children of average or higher negative affect, *b* = −0.11, 95% CI [−0.22, −0.01]. Examination of conditional indirect effects of family strains on symptom change via less responsive parenting showed statistical significance at average and higher levels of negative affect. Higher levels of family strains were also associated with higher levels of symptoms at grade 4, *b* = 0.45, *p* = 0.005, controlling for responsive parenting and child negative affect. Findings suggest that interventions for reducing risk for internalizing problems and promoting emotional health in children may show improved efficacy if modified to respond to family circumstances and stressors.

## 1 Introduction

Anxiety and depression, internalizing problems that are among the most prevalent mental health disorders diagnosed in children and adolescents (Center for Disease Control and Prevention [CDC] 2022), cause significant impairment in social, emotional, and academic functioning (Essau et al. 2014; Jaycox et al. 2009). Grade school represents a particularly sensitive developmental period for emotional disorders given children during this transitional period face new and more intense emotional demands as they navigate novel situations and responsibilities (e.g., conflict in peer friendships and more challenging academic tasks). Further, more structured schooling (e.g., more formal classroom rules and limitations on free play; longer school hours) can pose challenges for meeting novel academic expectations and managing self‐organization (Jiang et al. 2021). These major changes and new challenges co‐occur with relatively immature neurobiological systems and cognitive abilities necessary for self‐regulation (Morris et al. 2017). Despite this elevated risk, there is limited research on factors explaining variability in trajectories of anxious and depressive symptoms (see Papachristou and Flouri 2020, and Zdebik et al. 2019 for exceptions). In the present study, we examined whether greater family strains (e.g., financial strains, workplace stressors, financial resource deficits, lack of socioemotional support) undermine responsive parenting during preschool age, leading to elevations in anxiety and depressive symptoms in children following the transition into grade school, and whether this maladaptive pathway is more salient for children higher in temperamental negative affect.

### 1.1 The Family System and Child Internalizing Problems

Family processes have the potential to significantly impact the developmental course of depressive and anxiety symptoms due to the critical role of the family in aiding (or undermining) children's developing abilities to regulate difficult emotions. The *tripartite model* of children's emotion regulation and adjustment (Morris et al. 2007) highlights the various ways that the family context shapes the development of children's emotional health. In particular, when parents respond to child distress with warmth and patience, they serve as supportive emotional coaches, which help children learn and internalize more adaptive emotional responses (Morris et al. 2017). These parenting processes shape children's emotional regulation, a critical predictor of (fewer) emotional problems (Eisenberg and Morris 2002; Morris et al. 2007). Thus, it is not surprising that greater parental warmth toward preschoolers (age 5) has been linked to reduced risk of internalizing symptoms^1^ at age 11 (Kuhlman et al. 2014). Further, intervention studies have demonstrated how increases in parental warmth and sensitivity can have long‐term implications for children's internalizing symptoms (Yap et al. 2016). That said, although responsive parenting may play a key role in children's risk for anxiety and depressive symptoms, few studies have employed long term, longitudinal designs linking responsive parenting to trajectories of child internalizing problems during key developmental periods (Yap and Jorm 2015). Given increasing rates of internalizing disorders (CDC 2022; Zdebik et al. 2019), and the significant consequences associated with experiencing depressive and anxiety symptoms early in life (e.g., academic challenges, risk for psychopathology later in life; Essau et al. 2014; Jaycox et al. 2009), there is a critical need to understand how preexisting family processes impact trajectories of anxious and depressive symptoms as children navigate grade school.

There is also a critical need to study parenting in context, with particular attention to features of the larger family environment that impact parenting and directly shape children's emotional development (Morris et al. 2007, 2017). For example, Morris et al. (2007) highlight how the larger *family emotional climate* (e.g., quality of other relationships within the family, amount of positive and negative emotion that is displayed by family members; Bodovski and Youn 2010; Morris et al. 2007) impacts child emotion regulation. In negative family emotional climates, children may observe higher levels of emotion dysregulation in other family members or experience emotional coercion or manipulation, which undermines children's sense of emotional security and increases risk for emotional problems (Cummings and Davies 1996; Morris et al. 2007). In contrast, positive family emotional climates are better suited for meeting children's emotional needs and foster a strong sense of emotional security in children. Further, higher levels of dysfunction and negativity in the family have the potential to spill over into parenting, leading to less responsiveness and sensitivity which, as previously noted, are critical for healthy emotion socialization of the child (Morris et al. 2007).

Given the importance of a positive and supportive family emotional climate for promoting healthy child emotion regulation, broad risk factors for a more strained and dysregulated family environment represent prime candidates for understanding increased risk for child internalizing problems. Elevated stress is one of the most robust predictors of parental mental health and has the potential to undermine family functioning, leading to more adversarial dynamics and expressed negativity (Ingram and Luxton 2005; Schulz et al. 2004). For example, higher levels of workplace stress predict more expressions of irritability and anger in the home (Repetti et al. 2009). Further, this risk is not limited to the presence of overt stressors (e.g., conflict at work or in the family). Resource deficits can also negatively impact the family emotional climate. For example, economic pressure experienced by low‐income parents who lack financial resources contributes to strain and is associated with increased depressive affect in parents (Elder et al. 1995). Additionally, when families lack socioemotional resources (e.g., support), this strains the family system and leads to increased negativity and discord. Parents who report less access to social resources report more dysfunction across their family and workplace roles (Oren and Levin 2017; Sepa et al. 2004), which can lead to work–family conflict, a significant source of stress for parents (Oren and Levin 2017). Social resource deficits are also associated with risk for child maltreatment in the home (Paavilainen and Åstedt‐Kurki 2003) and less adaptive coping among children (Tariq and Majeed 2022). Thus, children from families experiencing more economic and social stressors and significant resource deficits (e.g., material or coping resources) are expected to be at greater risk for internalizing problems, in part due to these chronic family strains compromising responsive parenting.

### 1.2 Child Temperamental Negative Affect and the Family Context

Although specific parenting responses and the larger family emotional environment are fundamental to how children develop socioemotional abilities, child characteristics also play an important role. Indeed, development is conceptualized as an ongoing transaction between intrapersonal characteristics and interpersonal contexts (Granic and Hollenstein 2003). Most notably, child temperamental *negative affect* (NA) is characterized by intense and frequent expressions of negative emotions and a compromised ability to regulate arousal, which are key features of internalizing problems (Barańczuk 2019). Higher NA is associated with internalizing symptoms as early as preschool age (Crawford et al. 2011). Additionally, more recent research indicates children ages 2–3 with temperamental profiles high in NA show increased risk for elevated internalizing symptoms and diagnosis of an anxiety disorder at age 5 (Xie et al. 2022).

Although NA appears to predispose children toward developing internalizing symptoms, there is also evidence that this innate characteristic of the child interacts with the environment to determine outcomes. That is, not all children high in NA will inevitably develop anxiety and depression; this risk depends, in part, on whether they are also exposed to maladaptive parenting or lower levels of family cohesion (e.g., Morris et al. 2002; Oldehinkel et al. 2006; Rabinowitz et al. 2016). Nonetheless, research is inconclusive regarding the specific environmental conditions that interact with child NA to predict anxiety and depressive symptoms, and some studies even suggest counterintuitive patterns, whereby higher responsivity predicts *more* internalizing problems for children higher in negative affect (e.g., Davis et al. 2015; Kiff et al. 2011). Thus, it is imperative that researchers routinely account for the complex interplay between child NA and family processes when investigating risk for internalizing problems.

### 1.3 The Present Study

In the present study, we aimed to answer the following empirical questions: (a) Do broader family strains and specific parenting processes during preschool predict developmental trajectories of anxious and depressive symptoms across grade school? (b) Does less responsive parenting during preschool mediate the effect of elevated family strains on trajectories of anxious and depressive symptoms? and (c) Does child temperamental NA moderate these pathways? We predicted that greater family strains (e.g., financial strains, workplace stressors, financial resource deficits, lack of socioemotional support) would undermine responsive parenting during preschool age, leading to elevations in anxiety and depressive symptoms following the transition into grade school (four annual assessments spanning grades 1–4). Further, we predicted that this maladaptive family pathway would be more salient for children who are higher in temperamental NA given these children have demonstrated increased sensitivity to adversity (Morris et al. 2002; Oldehinkel et al. 2006; Rabinowitz et al. 2016).

The present study builds on prior work in several ways. First, we leveraged data from a large‐scale longitudinal investigation of 496 families, oversampling for economic risk and spanning two critical developmental stages—preschool and grade school. Altogether, this study spanned approximately 5 years over five waves of data collection. Second, we examined anxiety and depressive problems across a crucial developmental period in grade school, when children are at risk for escalating internalizing symptoms as they navigate increasing demands for autonomy and self‐regulation. Third, in preschool, prior to the transition to formal schooling, we implemented a comprehensive measure of chronic family strains comprising overt stressors (e.g., financial stress, workplace stress, neighborhood stress) and socioemotional resource deficits (e.g., lack of support from spouse or extended family, lack of supportive workplace environment)—strains that we conceptualized as contributing to a more negative emotional climate (i.e., more expressed negativity and family dysfunction) and undermining responsive parenting. An examination of family processes during preschool age is significant given this period is characterized by advances in cognitive abilities and socialization that allow children to engage in parent‐guided emotional regulation (Eisenberg and Morris 2002), laying a foundation for future emotional coping. Finally, we examined whether the preschool family environment, including responsive parenting and broader family socioemotional strains, differentially predicted anxiety and depressive symptoms as a function of child temperamental NA, which is critical for identifying children who are most sensitive to these family influences shaping risk for internalizing problems.

## 2 Method

### 2.1 Participants

The participants were 496 children (51.6% female) and their caregivers. Families were recruited from two US cities in the Midwest as part of a larger longitudinal study of cognitive development following a lagged cohort sequential design. All children were recruited when they were in preschool, and the majority were recruited when they were 3 years old. Smaller subsets of children were subsequently recruited when they were 3.75, 4.5, and 5.25 years of age. During grade school assessments, children's ages ranged from 6.39 to 8.37 years (*M* = 7.17, SD = 0.35) at grade 1; 7.26–9.40 years (*M* = 8.13, SD = 0.35) at grade 2; 8.29–10.41 (*M* = 9.11, SD = 0.36) at grade 3; and 9.26–11.29 years (*M* = 10.09, SD = 0.36) at grade 4. Initial recruitment techniques included flyer distribution in preschools and in other locations in the community. Eligibility criteria at study enrollment included English as the primary language spoken in the home. Other exclusion criteria included diagnosed developmental, behavioral, or language disorders at the time of study enrollment. Children diagnosed with a language, speech, or behavioral disorder *after enrollment* were retained in the study. Recruitment was stratified by sex and socioeconomic risk, with 55.2% of families meeting study criteria for increased socioeconomic risk (defined as enrollment in either public medical assistance or free school lunch programs, or meeting poverty criteria according to federal guidelines). The median household income for the sample was $37,400. The ethnic and racial makeup of the sample was representative of the region of recruitment, with 68.2% identifying as White, 5.6% as Black, 0.2% as Asian, 15.5% as multiracial, and 10.5% as Hispanic.

### 2.2 Procedures

At study entry, during preschool, research technicians conducted home visits. After obtaining consent from parents, research technicians administered a home observation measure. Parents also completed a questionnaire on perceived stressors and resource deficits. Approximately 1 week later, families attended an in‐lab session, where mothers completed a background interview and a questionnaire regarding their child's temperament. During the grade school phase of the study, families were asked to participate when children were in grades 1–4, at which point parents completed questionnaires rating their child's anxiety and depressive symptoms. Due to the cohort sequential design of the study and the timing of funding, participation varied across grade school assessments. For example, not all children were eligible to participate in the grade 1 assessment (i.e., some children were past grade 1), and at the end of the study, not all children had reached grade 4. At grade 1, 291 youth participated; 386 participated at grade 2; 402 participated in grade 3; and 391 participated at grade 4. Of the 496 families who participated in the preschool phase, 444 completed at least one assessment in the grade school phase (89.5%); 244 completed all four assessments; 125 completed three assessments; 45 completed two assessments; 30 completed one assessment; and 52 did not participate in this phase of the study. All procedures were approved by the Institutional Review Board at the University of Nebraska‐Lincoln.

### 2.3 Measures

#### 2.3.1 Family Strains

Parents completed the Life Stressors and Social Resources Inventory (LISRES; Moos and Moos 1994) at study entry to assess stressors and socioemotional resource deficits impacting the family unit *over the past year*. The LISRES includes 200 items answered in yes/no or Likert‐scale format. Items provide information about perceived stress or resource deficits across the following eight life domains: physical health; home and neighborhood; finances; work; spouse or partner relationships; relationships with children; relationships with extended family; and friends or social groups. Items assessing relational strains measure criticism, anger, unreasonable expectations, lack of empathy, and support deficits. Although some scales pertain more directly to strains experienced by a parent (e.g., work), other scales assess strains that are expected to impact all members of the family (e.g., home and neighborhood stressors; family relationship dysfunction; major life events such as the death of a family member).

In the present study, we computed an overall composite score of Chronic Family Strains. This is consistent with past research combining LISRES items reflecting stressors and resource deficits to create composites of overall family stress/strains (Wade et al. 1996; Yeates et al. 1997). First, we computed *T*‐scores for each subscale, based on published norms. Then, resource scores were reverse coded so that higher scores indicated greater resource deficits. Finally, subscale scores were averaged given not all scales applied to all families (e.g., work stress for unemployed parents). We did not include subscales that were specific to parenting stress (i.e., relationship with child) to ensure that the final score had adequate discriminant validity from the *responsive parenting* scale (described below) for a more conservative test of the association between family strains and responsive parenting. Therefore, all subscales, excluding the subscale related to the relationship with the child, were included. Cronbach's alpha across items included in the final composite (87 items with Likert ratings) was 0.77, suggesting adequate internal consistency.

#### 2.3.2 Responsive Parenting (Preschool)

The Early Childhood Home Observation for Measurement of the Environment (EC‐HOME; Caldwell and Bradley 1978) was conducted during preschool home visits to measure parent responsivity. In all cases, maternal responsivity was assessed. Research assistants were trained to 100% reliability by senior staff and regularly monitored to ensure administration fidelity. Items in the EC‐HOME pertaining to the Responsiveness Scale measure parents' emotional and verbal responsiveness to children and the overall warmth in the parent–child relationship. One item in the subscale is an interview item, while all other items assess the presence of target behaviors or parent–child interactions *that occur throughout the home visit*. The *responsiveness scale* assessed whether mothers held their children; conversed with their children; responded to children's questions; verbally acknowledged their children's comments; demonstrated spontaneous praise of their children; exhibited affection toward their children; and encouraged their children to show the interviewer an achievement. Interrater reliability in our studies using the EC‐HOME has been high (*κ* = 0.85–1.00). Raw scores for the Responsiveness Scale were used in the current study.

#### 2.3.3 Child Temperamental Negative Affect‐Very Short Form (Preschool)

Parents completed the Children's Behavior Questionnaire‐Very Short Form (CBQ‐Very Short; Putnam and Rothbart 2006). This version of the CBQ includes 36 items, and parents are asked to rate their children's temperament characteristics on a 7‐point Likert scale ranging from *extremely untrue* to *extremely true*. The CBQ‐Very Short includes the negative affect scale, which contains 12 items related to high anger, discomfort, sadness, fear, and difficulty soothing. Research indicates the CBQ‐Very Short has good psychometric properties (Putnam and Rothbart 2006). Internal consistency for negative affect scales in preschool ranged from 0.68 to 0.72 in this study. The first available score for negative affect in preschool was used in analyses.

#### 2.3.4 Child Anxiety and Depressive Symptoms (Grades 1, 2, 3, and 4)

Parents completed select subscales of the Child Behavior Checklist for Ages 6–18 (CBCL; Achenback and Rescorla 2001) when children were in grade school. Analyses in this study included the Anxious/Depressed scale, which consists of 13 items where symptoms are rated on a 3‐point scale (0 = *not true*; 1 = *somewhat or sometimes true*; 2 = *very true or often true*). Adequate internal consistency has been demonstrated for scales in the CBCL (*α* = 0.72 to *α* = 0.84; Achenbach 2001). Internal consistency for this sample ranged from 0.79 to 0.82 across grade school assessments. *T*‐scores were examined to determine whether scores reached clinical levels (i.e., *T*‐scores of 65 and above). Raw scores across grade school were used in analyses.

### 2.4 Data Analytic Plan

**FIGURE 1:**
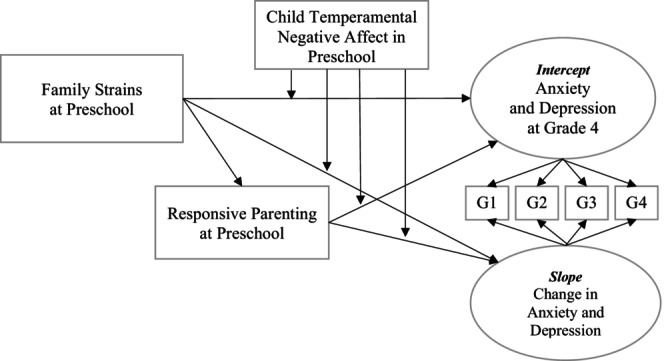
Conceptual model for moderated mediation analyses. We conceptualize family strains and a lack of socioemotional resources undermining responsive parenting, which predicts greater escalation in internalizing problems (i.e., anxiety and depressive symptoms) during grade school (slope) and greater problems at grade 4 (intercept) at higher levels of child temperamental negative affect. G1 = Grade 1, etc.

Data analysis was conducted using Mplus software v8 (Muthén and Muthén 2010). Full Information Maximum Likelihood (FIML) estimation was used to address missing data (Enders 2010). Missing data for at least one assessment in grade school was not significantly associated with race, ethnicity, or socioeconomic risk (defined as whether or not families received public medical assistance or free school lunch, or met poverty criteria based on federal guidelines). For the primary analyses, we used the MLR estimator which is robust to non‐normality; however, we transitioned to using ML with bias‐corrected bootstrapping for estimation of indirect effects. The conceptual framework guiding model specification is depicted in Figure 1. The CFI, TLI, RMSEA, and SRMR were computed to assess global model fit according to standard guidelines and thresholds (e.g., Bentler 1990; Browne and Cudeck 1993; Hu and Bentler 1999). Specifically, CFI/TLI values above 0.90 were interpreted as indicating acceptable model fit and above 0.95 were interpreted as indicating good model fit. RMSEA and SRMR values under 0.08 were interpreted as indicating acceptable model fit and values under 0.06 were interpreted as indicating good model fit. Child sex was assessed at enrollment and was used as a covariate in analyses.

Data analysis was completed in two stages. In stage 1, we tested a latent growth model with four equally spaced repeated measures at grade 1, grade 2, grade 3, and grade 4. Time was centered at grade 4 by fixing factor loadings for the slope factor to −3, −2, −1, 0 across the four repeated measures; this allowed us to model the intercept factor as anxiety and depressive symptoms at grade 4 in the predictive analyses. We compared three models reflecting different patterns of change over time: (a) intercept only (i.e., scores wax and wane; there is no systematic pattern of change), (b) linear model (i.e., there is a systematic linear pattern to the repeated scores), and (c) quadratic model (i.e., there is acceleration or deceleration in change over time). We evaluated Akaike's information criterion (AIC; Akaike 1987) and Bayesian information criterion (BIC; Sclove 1987), with smaller values reflecting the superior fitting model. We also conducted likelihood ratio tests to establish that a model (e.g., linear) significantly improved the fit relative to the more parsimonious nested model (e.g., intercept only).

In stage 2, we added predictors of the growth factors retained in stage 1. Specifically, growth factors were regressed on family strains, responsive parenting, child temperament, the interaction between family strains and temperament, the interaction between responsive parenting and temperament, and child sex (control variable). Further, responsive parenting (mediator) was regressed on family strains, child temperament, and child sex. All exogenous variables, including interaction terms, were covaried. The residual variances of the intercept and slope factors were also covaried as is customary in latent growth modeling. Scores of family strains, responsive parenting, and child temperament were standardized (i.e., *z*‐scored) prior to creating interactions to ease interpretation of simple slopes (i.e., conditional effects). We conducted a Johnson‐Neyman regions of significance analysis, which computes simple slopes at all levels of the continuous moderator (child temperament) and identifies at what levels of the moderator the predictor is related to the outcome (Hayes 2017). To test for mediation, a bootstrap approach (Shrout and Bolger 2002) was used for estimating indirect effects, including conditional indirect effects at levels of child temperament in the context of significant moderation. Bootstrapping provides an empirical approximation of sampling distributions of effects to produce confidence intervals (CI) of estimates. We used a nonparametric resampling method (bias‐corrected bootstrap with ML) with 20,000 resamples drawn to derive the 95% CIs for the indirect effects (Preacher and Hayes 2008).

## 3 Results

Descriptive statistics and correlations are reported in Table 1. Repeated measures of anxiety and depressive symptoms were highly correlated, as expected. Mean levels of anxiety and depressive symptoms across grade school were generally low (mean scores ranged from 2.63 to 3.13), with mean scores well under clinical levels (represented by a *T*‐score of 65 and raw score of 8). Clinical levels of symptoms were observed for 7.3% of the sample at grade 1; 7.6% at grade 2; 6.7% at grade 3; and 11.6% at grade 4. Family strains were significantly positively correlated with anxiety and depressive symptoms, indicating that more family strains were associated with more anxiety and depressive symptoms throughout grade school. A similar pattern was observed between measures of preschool temperamental NA and anxiety and depressive symptoms, suggesting children higher in NA exhibited more anxiety and depressive symptoms throughout grade school. Responsive parenting was only correlated with child anxiety and depressive symptoms during grade 2, and child sex was only correlated with anxiety and depressive symptoms at grade 4. Family strains were significantly associated with child negative affect such that parents of children with more NA reported greater family strains. Additionally, family strains and parent responsivity were significantly negatively correlated, showing that more strains in the family were associated with less responsive parenting. This correlation was also small in magnitude, demonstrating adequate discriminant validity of these family variables.

**TABLE 1:** Descriptive statistics and correlations for study variables.

	1	2	3	4	5	6	7	8
1. Anxiety/Depression, G1^a^	—							
2. Anxiety/Depression, G2	0.66**	—						
3. Anxiety/Depression, G3	0.62**	0.71**	—					
4. Anxiety/Depression, G4	0.64**	0.66**	0.71**	—				
5. Family strains	0.23**	0.23**	0.16**	0.21**	—			
6. Responsive parenting	0.07	0.09*	0.02	−0.02	−0.16**	—		
7. Child negative affect	0.21**	0.21**	0.22**	0.22**	0.22**	−0.05	—	
8. Child sex^b^	−0.04	−0.04	−0.05	−0.09*	−0.02	−0.00	−0.00	—
*M* (SD)	2.63 (3.12)	2.71 (3.22)	2.78 (2.99)	3.13 (3.35)	48.47 (5.16)	5.10 (1.33)	3.94 (0.77)	
*N*	291	386	402	392	493	496	496	

*Note:* Correlations between hypothesized predictors and child anxiety and depressive symptoms at each wave are shaded.

^a^ G1 = Grade 1, etc.

^b^ Child sex is coded 0 for female and 1 for male.

* *p* < 0.05.

** *p* < 0.01.

### 3.1 Stage 1: Growth Curve Models

Fit statistics for each model are reported in Table S1. Nested model comparisons using the Satorra‐Bentler Scaled chi‐square, in the context of MLR, suggested that the linear model was a superior fit to the nested intercept‐only model, *χ* ^2^ (3) = 10.50, *p* = 0.015; however, the quadratic model was not a significant improvement relative to the nested linear model, *χ* ^2^ (4) = 6.15, *p* = 0.188. The mean of the slope factor was positive and significant (*M* = 0.19, *p* < 0.001) suggesting that symptoms increased at a significant annual rate over time. The mean of the intercept factor was 3.08 suggesting that, on average, children had a score of 3.08 at grade 4, the last assessment occasion. Sixty‐six percent to 75% of the variance in the repeated measures was explained by the growth factors. The intercept factor showed significant variance, while the slope factor did not. Age at preschool assessment (in years) was not associated with the intercept (*b* = −0.03, *p* = 0.842) nor the slope (*b* = −0.002, *p* = 0.978) factors from the growth model and, accordingly, was not controlled for in subsequent analyses. Note that the growth modeling was conducted with a subsample of 444 children who had scores of anxiety and depressive symptoms at least once during grade school. Covariance coverage, reflecting the proportion of available data for pairs of variables (covariances) in the model, ranged from 0.56 to 0.84.

### 3.2 Stage 2: Moderated Mediation Model

Full model results are reported in Table 2. The global fit of the model was excellent (CFI = 0.994; TLI = 0.988; RMSEA = 0.021, 90% CI = 0.000, 0.048; SRMR = 0.020). Covariance coverage in the final model (*N* = 496) ranged from 0.50 to 1.00. Given that the *p* value for the interaction between responsive parenting and temperamental NA predicting the slope factor (i.e., changes in anxiety and depressive symptoms over time) was < 0.10 (*p* = 0.09), we probed the interaction to identify under what levels of child temperamental NA the simple slopes of parenting on changes in depressive and anxiety symptoms (slope factor) were significant. This approach is consistent with increasing recognition of the pitfalls of strictly adhering to conventional levels of statistical significance (e.g., the zero fallacy; Cumming and Calin‐Jageman 2016; Kline 2023). Results revealed that at around average levels of child NA (0.05 SD above the mean) and lower, the effect of responsive parenting on anxiety and depressive symptoms (simple slope), controlling for family strains and child sex, was not significant, *b* = −0.102, 95% CI [−0.21, 0.00]. However, at average and above levels of child NA (0.10 SD above the mean and higher), less responsive parenting during preschool was associated with greater escalation in symptoms from grade 1 to 4, *b* = −0.11, 95% CI [−0.22, −0.01].

**TABLE 2:** Model results for stage 2 analyses.

	Unstandardized coefficient	SE	*p*	Standardized coefficient (*r*)
Intercept: anxiety and depression at grade 4			*R*‐squared = 0.10	
Family strains	**0.45**	**0.16**	**0.005**	**0.16**
Responsive parenting	0.09	0.17	0.621	0.03
Child negative affect	**0.63**	**0.15**	**0.000**	**0.22**
Parenting × negative affect	−0.10	0.15	0.503	−0.04
Family strains × negative affect	−0.02	0.16	0.928	−0.01
Child sex	−0.48	0.31	0.116	−0.09
Slope: change in anxiety and depression from grade 1 to 4			*R*‐squared = 0.21	
Family strains	−0.05	0.05	0.396	−0.12
Responsive parenting	**−0.10**	**0.05**	**0.060**	**−0.27**
Child negative affect	0.03	0.06	0.602	0.08
Parenting × negative affect	**−0.09**	**0.05**	**0.088**	**−0.25**
Family strains × negative affect	−0.07	0.05	0.135	−0.19
Child sex	−0.11	0.10	0.286	−0.14
Responsive parenting at preschool age			*R*‐squared = 0.03	
Family strains	**−0.16**	**0.05**	**0.001**	**−0.16**
Child negative affect	−0.02	0.05	0.743	−0.02
Child sex	−0.01	0.09	0.913	−0.01
Exogenous covariances				
Sex‐negative affect	0.00	0.02	0.936	0.00
Sex‐family strains	−0.01	0.02	0.702	−0.02
Family strains‐negative affect	**0.22**	**0.05**	**0.000**	**0.22**
Endogenous residual covariances				
Intercept‐slope	0.51	0.33	0.127	0.56

*Note:* Bolded parameter estimates had *p* < 0.10 (double‐sided). Family strains, responsive parenting, and child temperamental negative affect were *z*‐scored.

**FIGURE 2:**
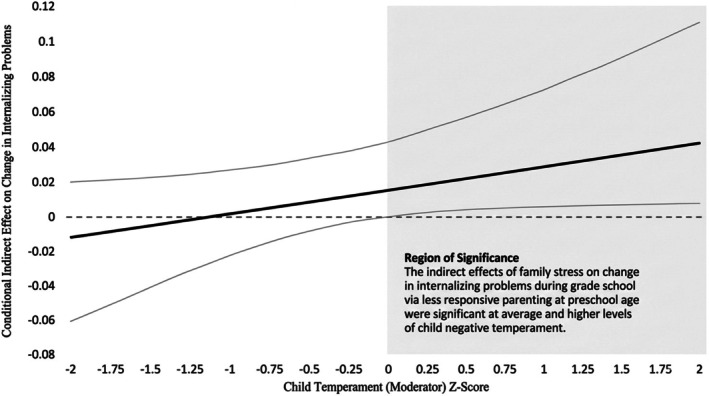
The conditional indirect effect of family strains on change in symptoms. The bold line represents conditional indirect effects of family strains on escalation in child anxiety and depressive symptoms (slope factor) via less responsive parenting at levels of child NA ranging from 2 SD below the mean to 2 SD above the mean. The 95% CI for the conditional indirect effect is represented by the light gray lines surrounding the bold line. Conditional indirect effects reached significance (i.e., the 95% CI did not contain zero) at average and higher levels of child negative temperament.

With regard to mediation pathways, higher levels of family strains were associated with less responsive parenting during preschool, *b* = −0.16, *p* = 0.001. Further, as just described, less responsive parenting was associated with greater escalation in anxiety and depressive symptoms over grade school for children of average or higher NA. As such, conditional indirect effects of family strains on the slope factor via responsive parenting at levels of child NA were estimated. The indirect effects of family strains on change in symptoms via less responsive parenting were significant (i.e., the bootstrapped 95% CI did not contain zero) at average and higher levels of child NA. See Figure 2 for a graphical depiction of the conditional effects at levels of child NA.

Responsive parenting did not interact with child NA to predict the *intercept* factor (i.e., grade 4 levels of anxiety and depressive symptoms), *b* = −0.10, *p* = 0.503, and the main effect of parenting on the intercept was not significant, *b* = 0.09, *p* = 0.621. A test of the indirect effect of family strains on grade 4 levels of anxiety and depressive symptoms through responsive parenting was not significant, *b* = −0.01, 95% CI [−0.07, 0.05].

Finally, we examined direct effects of family strains on the intercept and slope factors, in the context of the moderated mediation model. Higher levels of family strains were not associated with rates of change in anxiety and depressive symptoms over time (slope factor) but were associated with higher levels of anxiety and depressive problems at grade 4 (intercept factor). In the context of significant prediction of the intercept, but not the slope, we can conclude that the overall trajectory of anxiety and depressive symptoms was elevated to the extent that children were exposed to greater family strains during preschool, independent of responsive parenting, child NA, and child sex.

## 4 Discussion

In the present study, we investigated the effects of broad family strains on subsequent trajectories of child anxiety and depressive symptoms spanning grades 1–4, and examined whether unresponsive parenting was a key mechanism explaining this link. Further, because children who are higher in temperamental NA are at greater risk for developing internalizing problems and are more sensitive to environmental adversity (Morris et al. 2002), we investigated interactions between family pathways unfolding during preschool and child NA in predicting the course of child anxiety and depressive symptoms. Findings (i.e., significant annual increase from grade 1 to grade 4) align with prior research indicating that, on average, anxiety and depressive symptoms worsen over the course of childhood (Papachristou and Flouri 2020; Zdebik et al. 2019). Further, results indicate that more family strains predict greater escalation in child anxiety and depressive symptoms during grade school by undermining responsive parenting in preschoolers who exhibited average‐to‐high levels of NA.

It was notable that less responsive parenting during preschool emerged as a mechanism through which family strains impacted subsequent risk for maladaptive symptom trajectories. This provides evidence that greater family strains such as financial, workplace, and social stress, and lack of material and socioemotional supports have the potential to compromise a parent's ability to enact warm and nurturing behaviors toward their children, which is essential for healthy emotion socialization (Morris et al. 2017). In more chaotic family environments, parents report feeling more burdened and emotionally drained (Roy et al. 2004) and are less engaged in family‐focused activities and family routines (Fiese and Winter 2010)—critical opportunities for showing warmth and responsiveness to children. That said, although parent responsivity was associated with change in symptoms over time, it was not associated with symptom levels, even by grade 4, suggesting that responsive parenting during preschool might have a more subtle and protracted effect on trajectories of anxious and depressive symptoms, such that the ultimate impact of preschool parenting might not become apparent until later in development (e.g., during adolescence). That is, we might not observe the payoff of responsive parenting during preschool in terms of mitigating risk for anxiety and depression until later in development when children are even more autonomous and reliant on regulatory abilities that they have internalized as a result of skillful parenting. Nonetheless, future research is required to determine whether responsive parenting during preschool eventually predicts significant differences in levels of depressive and anxiety symptoms beyond the developmental period observed in the present study and, if so, at what specific age those differences emerge. It was also notable that this maladaptive familial pathway was only significant for children of average‐to‐high levels of temperamental NA. In contrast, lower NA children did not experience escalations in anxiety and depressive symptoms as a result of unresponsive parenting, perhaps due to fewer demands to downregulate negative emotions and less reliance on external regulators.

Although unresponsive parenting was a key pathway through which family strains impacted child risk for developing anxiety and depressive symptoms from grade 1 to grade 4, family strains also had a unique direct effect on this risk, independent of responsive parenting and child temperament, pointing to other unmodeled mechanisms tied to the emotional climate of the family (e.g., parental psychopathology, interparental conflict). Specifically, children had higher overall levels of anxiety and depressive symptoms from grade 1 to grade 4 to the extent that they were exposed to more family strains during preschool age. In contrast, there was no direct effect of family strains on rates of change in anxiety and depressive symptoms. This pattern of results suggests that family strains might have a more immediate and sustained effect on children's anxiety and depressive symptoms, with differences in symptom levels observed soon after children entered grade school and persisting thereafter (observed up to grade 4 in the present study). In the context of chronic family stress, children engage in more cognitive and behavioral avoidance (DeCarlo Santiago and Wadsworth 2009), coping strategies that can become more entrenched over time as these are favored over more active, problem‐focused strategies that are often ineffective in unpredictable environments (Wadsworth and Compas 2002), and greater reliance on avoidant coping is associated with increased depression and anxiety (Wadsworth 2015). Therefore, the sustained elevations in anxiety and depressive symptoms that were observed in the present study, as a result of exposure to more family strains, might reflect the limited coping repertoire children developed to adapt to a more negative early family environment.

### 4.1 Limitations, Future Directions, and Implications

There were several limitations to our study to consider when interpreting the findings. First, although our study draws from a theory addressing the development of emotion regulation in children (i.e., tripartite model), we did not directly measure emotion regulation in our study. We speculate that emotional regulation is a mechanism through which preschool family factors are associated with internalizing problems in later life. Future research, in which emotion development and regulatory skills are directly assessed, is necessary to further elucidate the developmental cascades through which early family characteristics impact child psychopathology. Further, despite using the tripartite model as a guiding framework, we did not use an explicit or comprehensive measure of the family emotional climate, which can encompass multiple indicators of emotional health in the family (Morris et al. 2007). Therefore, we look forward to future research using more direct measures of the family emotional climate in this line of work.

Second, we note methodological limitations. Our measurement of anxiety and depressive problems was limited to the first four grades, which we propose is a challenging phase for children given the increased emotional vulnerability children may experience as they navigate novel and more difficult tasks with immature regulatory systems. While our findings suggest that family factors present in preschool may have consequences for trajectories of anxiety and depression beyond fourth grade, research must examine outcomes into later childhood to determine how symptoms manifest in later developmental stages as a function of early family context. This extended observation period might also reveal greater individual differences in symptom trajectories for identifying risk and protective factors within the family system. While our sample represented diversity in socioeconomic status, our sample was relatively racially and ethnically homogeneous. It is critical to examine parallel research questions with families who face unique sources of family stress, such as persistent marginalization and stigmatization, to determine whether findings generalize. Further, there is an absence of research on how systemic racism impacts the family emotional climate, and the consequences of this for child development.

Additionally, the observed means of anxiety and depressive symptoms indicated few symptoms throughout grade school on average, with examinations of *T*‐scores indicating that 6.7%–11.6% of children exhibited clinical levels throughout grade school, with grade 4 showing the highest proportion. Therefore, findings could differ in a clinical sample of children with higher rates of diagnosable internalizing disorders. Low mean symptom levels and restricted range of symptoms could also be the result of using parent reports of child internalizing symptoms; internalizing problems can be more difficult to observe than other forms of psychopathology (e.g., externalizing problems). Future research might benefit from the use of child reports of internalizing problems when developmentally appropriate. Finally, the present investigation was focused on child temperamental NA given robust links with the development of depressive and anxiety symptoms; however, it is important to note that other temperamental traits can also interact with the family environment to explain psychopathology risk.

Despite these limitations, there are important implications of the present findings. Results inform the tripartite model of emotion regulation development (Morris et al. 2007) by providing evidence that broader family strains (i.e., stressors and resource deficits) play a critical role in a parent's ability to respond in a warm and sensitive manner to the child, which is critical for fostering healthy emotion regulation development. Indeed, our results suggest that a lack of responsive parenting during preschool, driven by chronic family strains, predicts higher levels of emotional problems across grade school for children who were predisposed to experiencing more negative emotions (i.e., high in temperamental NA). These findings also highlight preschool age as a critical developmental window for promoting family functioning that supports healthy emotion regulation development and suggest that it is critical to routinely account for child temperament to determine which children are most sensitive to family processes during key developmental phases. Indeed, children who were low in temperamental NA did not appear to experience negative outcomes in response to dysfunctional family dynamics.

Finally, early intervention is critical given the persistent nature of internalizing disorders, such as anxiety and depression, which tend to continue into later life when present in childhood (Cohen et al. 2018; Korhonen et al. 2018). Our study highlights the role of not only parenting, but also larger family influences, such as chronic family strains, on the development of anxiety and depressive symptoms. Further, results suggest that families navigating stress and adversity, in the absence of adequate resources, could benefit from interventions promoting responsive parenting during preschool age—especially for children high in NA—given this emerged as a key pathway through which the presence of greater family strains impacted trajectories of anxious and depressive symptoms. Yet, it was notable that family strains also demonstrated a unique, direct effect on anxious and depressive symptoms, suggesting that it might be insufficient to promote positive parenting alone and that interventions might be improved by addressing dysfunction in the broader family system.

## 5 Conclusion

Our findings indicate that preschoolers who are raised in families experiencing greater chronic strains (e.g., financial strains, interpersonal stressors, resource deficits) are at increased risk for maladaptive trajectories of anxiety and depressive symptoms spanning the first four school grades. Further, for children with above‐average temperamental negative affect, family strains lead to escalating symptoms due to less responsive parenting during a critical stage of child emotion regulation development. Results suggest that existing interventions that target parenting quality may demonstrate improved efficacy if modified to embrace a broader conceptualization of the family as a microsystem that is susceptible to strains. Chronic strains can contribute to a more toxic emotional climate that ultimately undermines healthy child development. Such interventions may be especially relevant for underrepresented and minoritized populations who experience a variety of chronic societal stressors known to impact individual health, as well as the health of the family system (Conger et al. 2010; Peverill et al. 2021).

## Funding

This work was supported by the National Institute of Mental Health (R01MH065668) and the National Institute of Diabetes and Digestive and Kidney Diseases (R01DK116693, R01DK116693‐04S1, R01DK125651).

## Conflicts of Interest

The authors declare no conflicts of interest.

## Supporting information


**Table S1:** famp70101‐sup‐0001‐TableS1.docx.

## Endnotes

^1^
Because symptoms of anxiety and depression are recognized as part of the *internalizing cluster* (Weersing et al. 2012) and are commonly assessed in research examining internalizing symptoms as outcomes, we present literature on internalizing symptoms more broadly, while focusing specifically on anxiety and depressive symptoms as outcomes of interest.
