# Supplementary material for: Navigating the Emotional Challenges of Grade School: The Influence of Early Family Strains and a Lack of Parental Responsiveness on Trajectories of Anxiety and Depressive Symptoms
Source: Fam Process. 2025 Dec 25;65(1):e70101. doi: 10.1111/famp.70101 (PMC12741509; doi:10.1111/famp.70101)
Supplement: Supplementary file 1 — Table S1: famp70101‐sup‐0001‐TableS1.docx. [file FAMP-65-0-s001.docx]

**Table S1**

*Global Fit Parameters for Intercept-only, Linear, and Quadratic Growth Models*

|  | AIC | BIC | χ^2^ (df) | Scaling Correction | CFI | TLI | RMSEA | SRMR |
| --- | --- | --- | --- | --- | --- | --- | --- | --- |
| Intercept-only | 6873.439 | 6898.013 | 18.70(8) | 1.60 | 0.97 | 0.98 | .055 | .051 |
| Linear | 6859.543 | 6896.406 | 7.03(5) | 1.42 | 1.00 | 0.99 | .030 | .029 |
| Quadratic | 6858.087 | 6911.333 | 0.56(1) | 0.96 | 1.00 | 1.00 | .000 | .006 |

*Note.* AIC = Akaike Information Criterion. BIC = Bayes Information Criterion. CFI = Comparative Fit Index. TLI = Tucker-Lewis Index. RMSEA = Root Mean Squared Error of Approximation. SRMR = Standardized Root Mean Squared Residual.
